# 

**Published:** 1890-09

**Authors:** 


### CONTENTS.

COMMUNICATIONS.

Tropho-Neurosis of the Oral Cavity with Especial Reference to Syphilitic Necrosis.... 421
Address of Dr. J. B. Patrick before the Southern Dental Association 431
Operative Dentistry.... 437
The Physiology and Pathology of the Peridentium.... 441
Evolution in Dentistry.... 446
Address to the Section of Dental and Oral Surgery.... 451
The Necessary Peroxide of Hydrogen.... 454
Clinical Observations on Operations for Congenital Clefts in Gums, Lips, and Palate in Early Infancy.... 456

SELECTIONS.

The Circulation of the Blood.... 464
Powerful Antiseptic.... 464

CORRESPONDENCE.

The Tenth International Medical Congress.... 465

EDITORIAL.

Personal.... 468
State Board of Dental Examiners.... 469
Ohio State Dental Society.... 469
Educational—The Dental Colleges.... 470
Personal—Removal.... 472

